# How to Grasp the Pelvis for Fixation in Contraction of the Hip

**Published:** 1880-02

**Authors:** 


How to Grasp the Pelvis for Fixation in Contraction of the Hip.—If a patient, with a contraction of the hip-joint, which is disguised by the oblique position of the pelvis, lies on his back on a level surface, the diseased leg touches with its posterior part the mattress, while the lumbar vertebræ are curved forward. If we now take hold of the healthy femur and bend it passively until it touches the chest, we see that the arch, which the lumbar vertebræ form above the mattress, flattens more and more as the flexion of the hip-joint increases. At last the column of the lumbar vertebræ is perfectly straight. At the same time the diseased femur rises from the mattress, and cannot be pressed down as long as the healthy femur is fixed. The cause of this is that the pelvis is fixed forcibly by aid of the passively flexed healthy leg. I shall only further point out that this position of the body may be of use both in *brisement force* and in gradual stretching of the hip-joint, whether we use passive manipulations or apparatus for extension. I was able to convince myself of the practicability of the method in question, both in *brisement force*, on account of contraction of the hip following coxitis, and in passive movements in cases of paralytic contraction with healthy hip-joint.

I have had no opportunity of trying whether this method may be of use by permanent extension. This might be done easily by fixing the flexed healthy femur, by aid of a broad band, while the diseased leg was permanently extended downward. Dr. R. Gersung in *Centralblatt fur Chirurgie.—Buffalo Med. and Surg. Journal.*

